# A comprehensive study of parameters correlated with honey health benefits†

**DOI:** 10.1039/d0ra10887a

**Published:** 2021-03-30

**Authors:** Aleksandar Marić, Pavle Jovanov, Marijana Sakač, Aleksandra Novaković, Miroslav Hadnađev, Lato Pezo, Anamarija Mandić, Nataša Milićević, Ana Đurović, Slobodan Gadžurić

**Affiliations:** Institute of Food Technology in Novi Sad, University of Novi Sad Bulevar cara Lazara 1 21000 Novi Sad Republic of Serbia pavle.jovanov@fins.uns.ac.rs; Institute of General and Physical Chemistry, University of Belgrade Studentskitrg 12-16 11000 Beograd Republic of Serbia; Faculty of Technology, University of Novi Sad Bulevar cara Lazara 1 21000 Novi Sad Republic of Serbia; Faculty of Sciences, University of Novi Sad Trg Dositeja Obradovića 3 21000 Novi Sad Republic of Serbia

## Abstract

One hundred honey samples of different floral origin (acacia, sunflower, meadow, and forest) collected from nine European countries (Serbia, Albania, Croatia, Montenegro, Romania, Bulgaria, Bosnia and Herzegovina, North Macedonia and Hungary) were analysed for various physicochemical, sensory, antioxidant and antibacterial parameters. The relative antioxidant capacity index and relative antibacterial index were calculated, integrated and expressed as a new property – *Power of Honey*, intended to be used to predict the health potential of a honey based on its antioxidant and antibacterial activities. Free acidity and colour coordinates L* and a* were chosen for building an artificial neural network model for the prediction of honey health potential. These were chosen based on the obtained correlations between the investigated parameters and in light of the simplicity of the analysis. This model successfully predicted the *Power of Honey* with a gained coefficient of determination of 0.856. Forest honey samples exhibited the highest *Power of Honey*. This novel approach should make it possible for honey producers to predict the honey health potential of a particular honey based on a quick and simple analysis.

## Abstract 2

One hundred honey samples of different floral origin (acacia, sunflower, meadow, and forest) collected from nine European countries were analysed for various physicochemical, sensory, antioxidant and antibacterial parameters.

## Graphical abstract

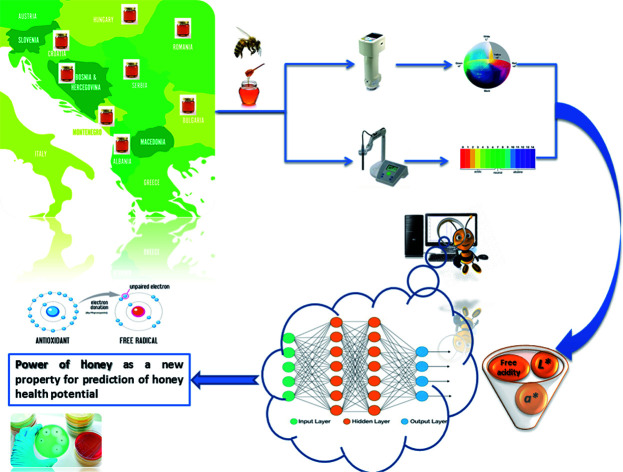

## Introduction

Honey is widely regarded as one of the first known functional foods. For centuries, if not millennia, it has been acknowledged for its beneficial health properties and as a ready-to-eat energy food.^1^ Even nowadays, in light of honey's medicinal properties, the scientific community continues to examine honey in attempts to discover any new benefits honey may possess. Among others, its antioxidant and antibacterial properties may indicate the practical potential of honey in the treatment of various diseases.

While the nutritional profile varies between different types of honey, mostly influenced by nectar, the secretions of flowering plants and/or excretions of plant-sucking insects, as well as by climate conditions and soil composition,^2^ the polyphenolic fractions are those elements which have been shown to be most responsible for observed differences in antioxidant activity.^3^ Flavonoids, phenolic acids, higher molecular weight polyphenols and carotenoids separately or synergistically modulate this activity. They do so by being incorporated into the cell membrane, thereby serving as fillers imparting rigidity to the membrane or as scavengers of free radicals involved in lipid oxidation.^4^ They also may diffuse in the cytosol, where they reduce the oxidative enzymes' activity and prevent the depletion of glutathione, protecting cellular organelles from damage by free radicals. Antioxidant activity can be estimated through chemical analysis, which measures different facets of an antioxidant's presence and impact, or with biological analysis, by measuring the protective effects of antioxidants against cellular damage by free radicals.^5^

The antibacterial properties of honey are highly complex, with several components documented as playing crucial roles in its potency, such as honey's high concentration of sugars, low pH value, high concentration of organic acids, bee defensin-1, glucose oxidase and polyphenolic compounds. Phenolic compounds, such as methyl syringate, provide honey with its ability to scavenge potent superoxide free radicals and, thus, exert its antibacterial activity.^6^

The measurement tools for estimating antioxidant or antibacterial potential are often based on different principles expressed in different units. Furthermore, they are often measured by different versions of the same assay in different laboratories. The inconsistencies in this process make comparisons of results complicated and often time-consuming. All these analyses require the utilisation of a particular methodology.

While honey's health benefits may be accredited to its antibacterial or antioxidant properties, the essential question for honey consumers is what is a honey's total potential, a single measure that would indicate the united potency of the antibacterial and antioxidant properties. In order to estimate this “joint property,” defined as *Power of Honey*, and its relations with physicochemical and sensory properties, different types of honey were investigated in this study. Since honey health potential is derived to a certain extent from its combined activities, the goal of this research was to predict various honey's health potential by (1) combining the results of antioxidant and antibacterial properties of different honey types (acacia, sunflower, meadow, and forest) to obtain the *Power of Honey* and (2) determining how and to what extent the *Power of Honey* is related to the examined physicochemical and colour properties of honey (principal component analysis – PCA and artificial neural network – ANN). The selection of parameters/analyses was made according to their level of correlation to the *Power of Honey* and the relative ease with which examinations of these elements could conducted.

## Material and methods

### Collection of samples

One hundred honey samples (25 meadow, 25 acacia, 25 forest and 25 sunflower) harvested in 2018–2019 from 9 countries (Serbia, Albania, Croatia, Montenegro, Romania, Bulgaria, Bosnia and Herzegovina, North Macedonia and Hungary) were used in this study. Approximately 1 kg of each honey sample was obtained from collectors and kept in the dark at room temperature until analysis.

### Moisture content

The moisture content was measured according to the methods of AOAC^7^ using a refractometer (Schmidt/Haensch ATR-ST, Lab exchange – Die Lab orgerätebörse GmbH, Burladingen, Germany).

### Acidity

A solution containing 10 g of honey in 75 mL of CO_2_-free distilled water was prepared for free acidity determination.^7^ The titration was done using 0.1 M NaOH, yielding a pH of 8.5. The acidity was calculated by multiplying the volume of 0.1 M NaOH needed for neutralisation by 10 and expressed in meq kg^−1^.

### pH measurement

The solution containing honey in water (20% w/v) on a dry matter basis was prepared for pH measurement. The moisture content of a honey sample determined by the refractometer (described in Moisture content) was used to calculate the correct dilution. The measurement was done using a SevenEasy™ pH meter (Mettler Toledo, Urdorf, Switzerland) and InLab 427 electrode (Mettler Toledo). The pH standards of 4.01 ± 0.02 and 7.01 ± 0.02 were used for calibration before each measurement.^8^

### Electrical conductivity

A RIAC conductivity meter CM 100/E with an YSI 3418 electrode (Yellow Springs Instruments Inc., Yellow Springs, OH) was used to determine electrical conductivity.^8^ The measurement was done using the solution previously prepared for pH determination (described in section pH measurement).

### Glucose and fructose analysis

Determination of glucose and fructose in honey samples was done according to the HPLC method described by Sakač *et al.*^9^ HPLC analysis was carried out using a liquid chromatograph (Agilent 1200 series, Agilent Technologies Inc., USA), equipped with an evaporative light scattering detector (ELSD), on an Agilent, Zorbax Carbohydrate 4.6 × 250 mm, 5 μm column (Agilent Technologies, USA) with acetonitrile and water (75 : 25, v/v) as a mobile phase. The flow-rate was 1.10 mL min^−1^ and the total run time was 12 min.

### Hydroxymethylfurfural (HMF) analysis

#### Sample preparation

The extraction procedure was described by Sakač *et al.*^9^ based on the method of Rufián-Henares *et al.*^10^ with some modifications made by Petisca *et al.*^11^

#### HPLC-DAD analysis

The determination and quantification of HMF were done using the HPLC method described by Ariffin *et al.*^12^ and Tomasini *et al.*^13^ with some modifications. HPLC analysis was performed using a liquid chromatograph (Agilent 1200 series, Agilent Technologies Santa Clara, CA, USA) equipped with a DAD detector and an Eclipse XDB-C18, 1.8 μm, 4.6 × 50 mm column (Agilent). The column temperature was 30 °C, and the sample injection volume was 2 μL. The mobile phase consisted of two eluents, H_2_O (0.1% HCOOH) (A) and methanol (B). The flow rate was 0.75 mL min^−1^. The isocratic elution was applied at the ratio A : B (90 : 10, v/v). The total run time was 5 min.

### Colour

The colour of the honey samples was measured using a Minolta Chromameter (Model CR-400, Minolta Co., Osaka, Japan) to obtain CIE L*a*b* coordinates. 20 mm-thick holders were used for samples (20 g), whose colour was measured against a black-and-white background at room temperature (23 ± 1 °C). The colour measures for each sample were taken at five points (1 central and 4 corner points). Measurements were done in 25 replications.

### Mineral analysis

The mineral content in honey was determined by the AAS method described by Sakač *et al.*^9^ The total mineral content was expressed as a sum of all determined minerals (K, Na, Ca, Mg, Fe, Cu and Zn).

### Total polyphenolic content

To determine total phenolic content (TPC), the Folin–Ciocalteu method described by Ferreira *et al.*^14^ was used, with some modifications. Each honey sample (1 g) was dissolved in 20 mL of distilled H_2_O. The honey solution (8 mL) was then used and mixed with 500 μL of diluted Folin–Ciocalteu reagents (1 : 10) for 3 minutes, followed by an addition of 1.5 mL of 25% sodium carbonate. The mixture was shaken and left to stand in the dark at 25 °C for 2 hours. The absorbance was measured at 750 nm against the blank using a spectrophotometer (Specord M40, Carl Zeiss, Jena, Germany). Gallic acid (1.25–31.25 μg mL^−1^) was used as the standard for the construction of the calibration curve, and the total phenolic content was expressed as gallic acid equivalents (GAE) (mg GAE/100 g of honey).

### Total flavonoid content

Each honey sample (0.5 g) was dissolved in 1 mL of distilled H_2_O. The honey solution was mixed with 300 μL of 5% NaNO_2_. After 5 minutes, 300 μL of 10% AlCl_3_ was added, and the mixture was vortexed. After 6 minutes, the solution was neutralised with the addition of 2 mL of NaOH (1 M). The absorbance was measured at 510 nm against the blank using a spectrophotometer (Specord M40, Carl Zeiss, Jena, Germany). Catechin (1.25–50.0 μg mL^−1^) was used as the standard, and the total flavonoid content was expressed as catechin equivalents (CAE) (mg CAE/100 g of honey).^15^

### Total carotenoid content

Total carotenoid content (TCC) extraction was carried out using the method previously described by Ferreira *et al.*^14^ with some modifications. Two grams of each sample was shaken with 10 mL of *n*-butanol saturated with H_2_O. The sample was then left to stand in the dark at room temperature for 18 h. Consequently, the sample was mixed and filtered through a filter paper (Whatman, Grade 4 Chr, UK). The absorbance was measured at 436 nm in comparison to the blank using a spectrophotometer (Specord M40, Carl Zeiss, Jena, Germany). Beta-carotene was used for the construction of the calibration curve (0.24–3.84 μg mL^−1^). The total carotenoid content was expressed as mg of β-carotene equivalents (BCE) (mg BCE/kg of honey).

### DPPH radical-scavenging activity

The scavenging activity of honey against 1,1-diphenyl-2-picrylhydrazyl radicals (DPPH˙) was estimated according to the procedure described by Hatano *et al.*,^16^ with some modifications. A honey sample (2 g) was dissolved in 10 mL of distilled water, centrifuged (3000×*g*) and filtered. Then, 0.1 mL of each of the various concentrations of the honey solution (25.0, 50.0, 100, 200, 400, and 800 mg mL^−1^) was diluted in 2.9 mL of methanol followed by an addition of 1 mL of a 90 μmol L^−1^ methanol solution of DPPH. The control was done with distilled water instead of a honey solution. The reaction mixtures were vortexed and left to stand in the dark at room temperature for 60 min. The absorbance was measured at 517 nm against the blank using a spectrophotometer (Specord M40, Carl Zeiss, Jena, Germany). The IC_50_ value (mg mL^−1^) was defined as the concentration of honey which was required to neutralize 50% of the initial amount of DPPH˙.

### Antibacterial activity

Honey solutions were prepared by diluting honey immediately before analysis to obtain a range of dilutions (37.5%, 18.7%, 9.3%, 4.6%, and 2.3%). Consequently, the samples were incubated in the dark at 30 °C for 30 min. For the antibacterial activity assays, the following Gram-negative bacteria were used; *Escherichia coli* (ATCC 11229), *Escherichia coli* I (clinical strain), *Pseudomonas aeruginosa* (ATCC 35554), and *Proteus mirabilis* I (clinical strain); and the following Gram-positive bacteria; *Staphylococcus aureus* (ATCC 6538), *Staphylococcus aureus* I (clinical strain), *Bacillus subtilis* (ATCC 6633), and *Enterococcus faecalis* (ATCC 19433).

Microdilution analysis was performed to determine the minimal inhibitory concentration (MIC). Pure bacterial strains were subcultured on nutrient agar slants at 37 °C for 24 h, while suspensions of the tested strains corresponded to the McFarland 0.5 optical density ≈ 1.5 × 10^8^ CFU mL^−1^. Each sample (50 μL) was added to 50 μL of Műeller Hinton Broth (Himedia, India) seeded with 1 μL of bacterial suspensions. The microtiter plates were incubated at 37 °C for 24 h and shaken. The MIC of the samples was determined following the addition of 10 mL of 2,3,5-triphenyl tetrazolium chloride (1% solution) and an incubation at 37 °C for 2 h, until the development of the red colour.

The controls for plate sterility and bacterial growth without samples were also included. The lowest concentration of solutions that inhibited bacterial growth, which was identified by the absence of red formazan, was considered to be the MIC. The MIC determination was performed in three replicates and three independent experiments.^17^

### Determination of relative antioxidant capacity index (RACI) and relative antibacterial index (RAI)

Central tendency is the most widely used method for comparing the antioxidant activity of complex food samples determined using multiple analyses.^18^ The samples are ranked according to the min–max normalisation, using the extreme values obtained from the analyses. The data in each set were transformed into normalised scores according to the following equations:1
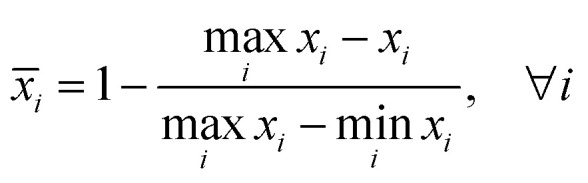
in cases of “the higher, the better” criteria (used for phenols, flavonoids and carotenoids content) (1)or2
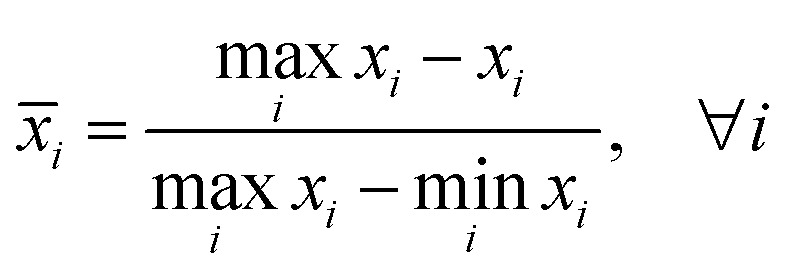
in cases of “the lower, the better” criteria (used for DPPH and MIC values), *x* represents the raw data.

The average of the standard scores obtained from the different analyses for a specific sample yields a single unitless value; the relative antioxidant capacity index (RACI). This same principle was applied in calculating the relative antibacterial index (RAI).

### Statistical analyses

Statistical processing of the data was carried out using the software package STATISTICA 10.0 (StatSoft Inc., Tulsa, OK, USA). Results were presented as mean ± standard deviation of the triplicate analyses for all measurements, excepting the colour determination of the samples, which was performed in 25 repetitions.

Principal component analysis (PCA) was used to discover the possible correlations among measured parameters and to classify objects.

### Artificial neural network (ANN) modelling

A multi-layer perceptron model (MLP) consisting of three layers (input, hidden and output layers) was used for model establishment.^19^ Prior to calculation, both input and output data were normalised to improve the ANN's behaviour. The Broyden–Fletcher–Goldfarb–Shanno (BFGS) algorithm^20^ was used as an iterative method for resolving unconstrained nonlinear optimisation problems in the ANN modelling.

The ANN experimental database was randomly divided into training, cross-validation, and testing data (with 60%, 20% and 20% of experimental data, respectively). The training data set was used for the learning cycle of the ANN and for evaluating both the optimal number of neurons in the hidden layer and the weight coefficient of each neuron in the network.

Coefficients associated with the hidden layer were grouped in matrices *W*_1_ and *B*_1_, while coefficients associated with the output layer were grouped in matrices *W*_2_ and *B*_2_. The neural network is typically presented using matrix notation (*Y* is the matrix of the output variables, *f*_1_ and *f*_2_ are transfer functions in the hidden and output layers, respectively, and *X* is the matrix of the input variables):^21^

3 *Y* = *f*_1_(*W*_2_*f*_2_(*W*_1_*X* + *B*_1_) + *B*_2_)

The elements of matrices *W*_1_ and *W*_2_ were determined during the ANN learning cycle, in the iterative procedure, using an optimisation algorithm to minimise the error between network outputs and experimental results.^22^ The coefficients of determination were used as parameters to check the performance of the obtained ANN model.

### Sensitivity analysis

Sensitivity analysis was used to examine the effects of the uncertainties in the observed parameters of the developed model.^23^ Neural networks can approximate experimental results, which can be partially noisy and/or contain partially imprecise data, and, therefore, sensitivity analysis is necessary to check if the neural network could behave erroneously.^24^ Therefore, infinitesimal amounts (+0.0001%) were added to each input variable, in 10 equally spaced individual points encompassed by the minimum and maximum of the training data.

## Results and discussion

This study aimed to examine the physicochemical, colour, and antioxidant and antibacterial property parameters of honey (Tables S1–S4†), and to identify their correlations (Table S5†), with the purpose of gaining insight into the honey health properties expressed as the *Power of Honey*. An artificial neural network model was employed in an attempt to predict the *Power of Honey*, calculated as the average of the relative antioxidant capacity index (RACI) and the relative antibacterial index (RAI).

### Correlations between the physicochemical, colour, and antioxidant and antibacterial property parameters of honey

For the estimation of the antioxidant potential, the content of polyphenols, flavonoids and carotenoids were determined, and DPPH radical scavenging activity was measured (presented in Table S3†). Positive correlations were observed between polyphenols and flavonoids (*r* = 0.7135; *p* < 0.001), while negative correlations existed between polyphenols or flavonoids and DPPH radical scavenging activity (*r* = −0.3187 and *r* = −0.3863; *p* < 0.001 (Table S5†). Similar correlations of polyphenols and flavonoids with DPPH radical scavenging activity have also been observed in honey from Tunisia,^25^ Turkey^26^ and Brazil.^27^ The results of antibacterial activity are presented in Table S4.† Among the examined compounds which were hypothesized to be potentially responsible for antibacterial activity, negative correlations were observed between the content of polyphenols and the suppression of the following Gram-negative bacteria; *Escherichia coli* (*r* = −0.5278; *p* < 0.001) and *Escherichia coli* I (*r* = −0.3815; *p* < 0.001), and the following Gram-positive bacteria; *Staphylococcus aureus* I (*r* = −0.3408; *p* < 0.001), *Bacillus subtilis* (*r* = −0.4367; *p* < 0.001) and *Enterococcus faecalis* (*r* = −0.2113; *p* = 0.035) (Table S5†). A negative correlation between phenolic content and antibacterial activity was also documented for honey samples collected from Morocco.^28^ Moreover, statistically significant correlations were observed between flavonoid and carotenoid content and the suppression of *Pseudomonas aeruginosa* (*r* = −0.3925 and *r* = −0.6175; *p* < 0.001). The content of carotenoids was also observed as negatively correlated with the suppression of *Proteus mirabilis* I (*r* = −0.5491; *p* < 0.001). Also, good correlations between DPPH radical scavenging activity and the suppression of *Escherichia coli* I (*r* = 0.6021; *p* < 0.001) and *Staphylococcus aureus* I (*r* = 0.6156; *p* < 0.001) (Table S5†) were observed.

Various physicochemical (Table S1†) and colour parameters (Table S2†) were studied to investigate their relationships with antioxidant (Table S3†) and antibacterial activities (Table S4†) and their correlations (Table S5†).

Statistically significant correlations (*p* < 0.001) were found between the free acidity of investigated honey samples and polyphenols (*r* = 0.7068), flavonoids (*r* = 0.7864), DPPH radical scavenging activity (*r* = −0.4752), *Bacillus subtilis* (*r* = −0.4890), *Staphylococcus aureus* (*r* = −0.4979), *Escherichia coli* (*r* = −0.4705) and *Escherichia coli* I (*r* = −0.4119) (presented in Table S5†). Total polyphenol content was shown to be correlated with the L* and b* honey colour coordinates (*r* = −0.4534, *r* = 0.4952; *p* < 0.001, respectively). Flavonoid content was also shown to be closely correlated with these same parameters (*r* = −0.8305, *r* = 0.7395; *p* < 0.001, respectively), a result previously observed in honey samples from Malaysia.^29^ Strong correlations were observed between DPPH radical scavenging activity and the a* (*r* = −0.7153; *p* < 0.001) and b* colour parameters (*r* = −0.5708; *p* < 0.001) (Table S5†). These findings are in line with the results of research carried out on Tunisian honey.^25^

Electrical conductivity was shown to be governed by the content of minerals (*r* = 0.6285; *p* < 0.001), which were determined to vary according to the floral origin^30^ and ionization products of the present free organic acids (*r* = 0.6694; *p* < 0.001) (Table S5†). A higher content of polyphenols was shown to promote ionization of the indigenous organic acids (*r* = 0.7068; *p* < 0.001), leading to stronger chelation of metals (*r* = 0.5178; *p* < 0.001) and concomitant potentiation of the antioxidant activity of polyphenolic compounds (Table S5†).

### Selection of ANN model parameters

The absence of any statistically significant correlation between the glucose and fructose content and the antioxidant activity parameters indicates that sugar content had no observable effect on this activity. However, a significant correlation was observed between glucose content and antibacterial activity against *Enterococcus faecalis* (*r* = 0.2235; *p* = 0.025) and *Escherichia coli* I (*r* = 0.2026; *p* = 0.043), as well as between fructose content and antibacterial activity against *Pseudomonas aeruginosa* (*r* = 0.3464; *p* < 0.001). Moisture and HMF content were not shown to exert a statistically significant impact on antioxidant or antibacterial parameters. This finding supports the claim that the formation of HMF does not influence changes in antioxidant activity, as was found in analyses of different types of Polish honey, excepting linden tree honey.^31^

In establishing the physicochemical and colour parameters for building a statistical model that could enable the relatively simple prediction of honey antioxidant and antibacterial properties, *e.g.* the combined parameter *Power of Honey*, particular consideration was given to: the availability of instruments for analysing honey samples, the time needed for analysis, and the costs involved. The analysis of free acidity and the measurement of honey colour coordinates (L* and a*) were chosen as the foundation elements for creating the ANN model.

### The common action of RACI, RAI and the *Power of Honey*

**Fig. 1:**
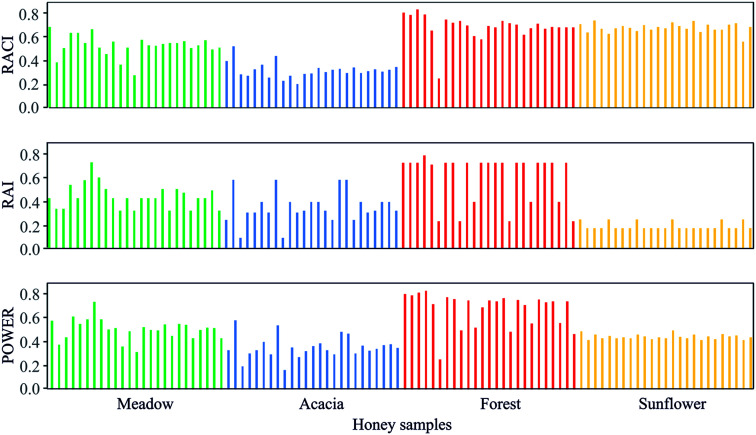
Relative antioxidant capacity index (RACI), relative antibacterial index (RAI) and the *Power of Honey* for the investigated honey samples.

When choosing between different types of honey, the honey consumer likely wants clear and concise information about the honey's potential health properties. The *Power of Honey* parameter was developed precisely for the purpose of enabling producers to provide such information to consumers. In this study, distinctions in health properties were observed according to the different floral origins of honey, with forest honey clearly standing out as more potent than the other honey types (Fig. 1).

### PCA analysis

**Fig. 2:**
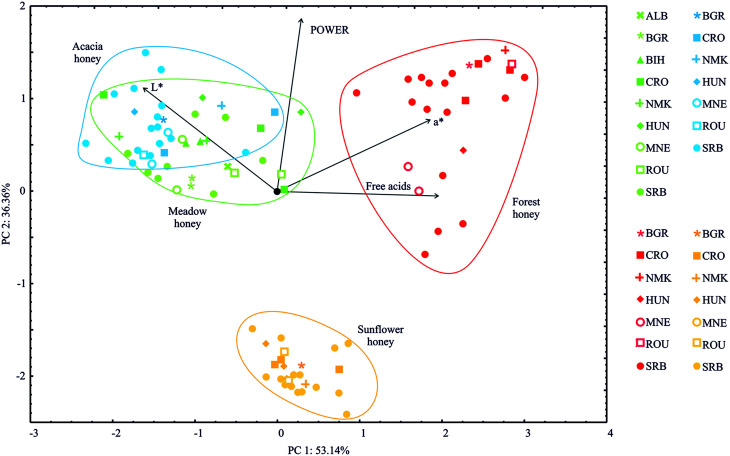
PCA ordination of variables based on component correlations of the free acids, colour coordinates L* and a*, and the *Power of Honey* samples.

Fig. 2 presents the PCA (principal component analysis) of four honey types (two monofloral – acacia and sunflower and two polyfloral – meadow and forest) collected in nine European countries. The PCA of the presented data demonstrated that the first two components accounted for 89.49% of the total variance (53.14% and 36.36%, respectively) for four variables (free acids, the colour coordinates L* and a* and the *Power of Honey*). The PCA map performed on the data indicated that free acids (which explained 38.1% of the total variance, based on correlations) and the a* colour coordinate (33.9%) exhibited positive scores according to the first principal component. In contrast, the L* colour coordinate (27.2%) displayed negative score values according to the first principal component (Fig. 2). Regarding calculations of the second principal component, a positive correlation was observed for the L* colour coordinate (23.4% of the total variance, based on correlations) and the *Power of Honey* (65.1%).

The highest a* colour coordinate, free acidity, and *Power of Honey* indicators were observed in the forest honey samples, as shown in Fig. 2. Higher L* values were found for acacia and meadow honey samples.

The PCA categorised all the examined honey samples into four groups, corresponding to the honey samples' floral origin. The sunflower and forest honey samples were isolated to form distinctive clusters, whereas the acacia and meadow honey samples could not be separated based on the examined physicochemical parameters.

### ANN model

The acquired optimal neural network model showed a good generalisation capability for the experimental data and could predict the *Power of Honey* accurately. According to the ANN performance, the optimal number of neurons in the hidden layer for the *Power of Honey* calculation was 10 (network MLP 3-10-1) for obtaining high values of *r*^2^ (overall 0.856) and low values of *SOS* (Table 1). In the table, the term *Performance* shows the coefficient of determination, while the term *Error* indicates a lack of data for the ANN model.

**Table 1:** Artificial neural network model summary (performance and errors) for training, testing and validation cycles^a^

Network	Performance			Error			Train. algor.	Error function	Hidden activation	Output activation
	Train.	Test	Valid.	Train.	Test	Valid.				
MLP 3-10-1	0.856	0.941	0.857	0.003	0.001	0.003	BFGS 12	SOS	Logistic	Logistic

a Here, the term performance represents the coefficients of determination, while error terms indicate a lack of data for the ANN model; train. – training cycle; test – testing cycle; valid. – validation cycle; SOS – sum of squares.

The ANN model predicted experimental values for the *Power of Honey* reasonably well for a broad range of the free acid values and for the colour coordinates L* and a*. *SOS* values obtained with the ANN model were of the same order of magnitude as experimental errors previously reported in relevant literature.^32^ The ANN model is complex (51 weights-biases) because of the developed system's high nonlinearity.^33^Table 2 presents the elements of matrix *W*_1_ and vector *B*_1_ (presented in the bias row), while Table 3 presents the elements of matrix *W*_2_ and vector *B*_2_ (bias) for the hidden layer used for calculation in eqn (3). The ANN model displayed an insignificant lack of fit tests, signifying that the model satisfactorily predicted the *Power of Honey*. The high *r*^2^ was an indication that the variation was accounted for and that the data fitted the proposed model satisfactorily.

**Table 2:** Elements of matrix *W*_*1*_ and vector *B*_*1*_ (presented in the bias column)

	1	2	3	4	5	6	7	8	9	10
L*	−5.138	13.497	4.866	21.350	7.121	6.577	2.363	3.858	22.490	6.662
a*	−3.305	8.452	2.709	13.619	4.459	4.378	−0.368	3.155	14.139	4.294
Free acidity	0.015	−0.248	0.105	−0.531	−0.236	−0.139	−0.312	−0.225	−0.741	−0.243
Bias	0.038	−0.032	0.251	−0.054	−0.253	0.176	1.099	−0.564	−0.196	0.045

**Table 3:** Elements of matrix *W*_2_ and vector *B*_2_ (presented in the bias column)

	1	2	3	4	5	6	7	8	9	10	Bias
*Power of Honey*	1.716	−12.843	−29.156	−28.476	−15.307	43.337	−3.580	10.276	28.519	12.963	−4.963

### Sensitivity analysis

**Fig. 3:**
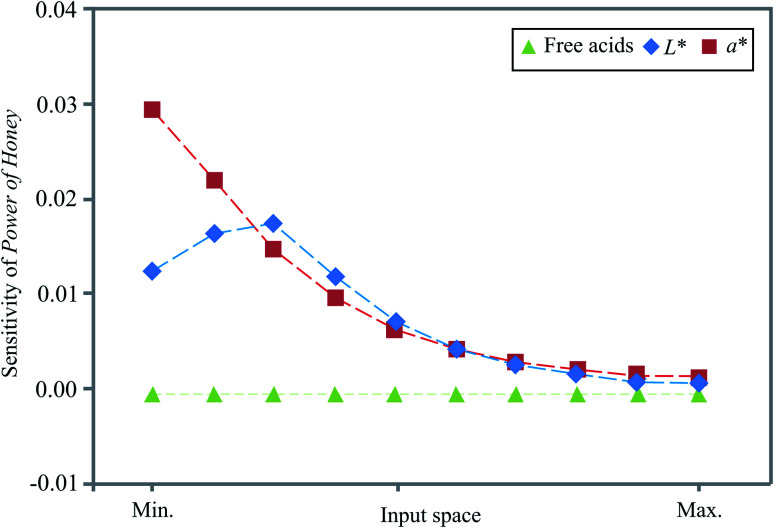
Sensitivity analysis.

In this study, the input variables' influence at a specific position in the input space over the output variables was tested using sensitivity analysis. Sensitivity values are the first-order derivatives evaluated at the specific centile points of each input variable. For each input, the derivative was taken in relation to the target at ten evenly spaced locations in the range of the observed minimum and maximum values. The exact values were calculated using the Taylor formula.^34^Fig. 3 displays the influence of the input over the output variables, *i.e.* the calculated changes of output variables for infinitesimal changes in input variables, as well as the importance of an input variable at a given point in the input space. The obtained values corresponded to the level of experimental errors and demonstrate how free acid values and the L* and a* colour coordinates influenced the *Power of Honey*.

As shown in Fig. 3, small changes in the colour coordinates L* and a* positively affected the *Power of Honey* throughout the input space, most intensively at the minimum (a*) and in the middle (L*) of the input space.

## Conclusion

In this study, the relationships between the physicochemical, sensory, antioxidant and antibacterial property parameters of different honey types from nine European countries were investigated. The results of the antioxidant and antibacterial activities were combined to obtain a parameter defined as the *Power of Honey*, which could be used to predict the health potential of a particular honey. Based on the obtained correlations and taking the simplicity of the investigated analyses into account, free acidity and the colour coordinates L* and a* were chosen as the basic factors for building the artificial neural network model for predicting honey health potential, expressed as the *Power of Honey*. The ANN model successfully predicted the *Power of Honey* with an obtained coefficient of determination of 0.856.

The obtained ANN model results showed that the forest honey samples exhibited the highest *Power of Honey* values (compared to acacia, meadow and sunflower honey samples), indicating that these samples exhibited the highest health potential. The developed ANN model can be applied in initially examining the health potential honey using simple analyses.

## Author contributions

Aleksandar Marić, Pavle Jovanov, Marijana Sakač and Lato Pezo participated in the conception and design of the study. Lato Pezo and Ana Đurović were responsible for the acquisition of data. Aleksandar Marić, Pavle Jovanov, Aleksandra Novaković, Miroslav Hadnađev and Nataša Milićević participated in the analysis and interpretation of data. Preparation of the manuscript was performed by Aleksandar Marić and Pavle Jovanov, while Marijana Sakač, Slobodan Gadžurić and Anamarija Mandić critically revised the manuscript for important intellectual content.

## Conflicts of interest

There are no conflicts to declare.

## Supplementary Material

RA-011-D0RA10887A-s001

## References

[cit1] Jaafar K., Haidar J., Kuraydiyyah S., Ghaddar T., Knio K., Ismail B., Toufeili I. (2017). J. Food Sci. Technol..

[cit2] Codex Alimentarius Commission , Revised Codex Standards for Honey. Codex Standard 12–1981, Rev. 2, 2001

[cit3] da Silva P. M., Gauche C., Gonzaga L. V., Costa A. C., Fett R. (2016). Food Chem..

[cit4] Nespolo M. , Free radicals in biology and medicine, ed. B. Halliwell and M. C. J. Gutteridge, Oxford University Press, 5th edn, 2015

[cit5] Salonen A., Virjamo V., Tammela P., Fauch L., Julkunen-Tiitto R. (2017). Food Chem..

[cit6] Almasaudi S. B., Al-Nahari A. A. M., Abd El-Ghany E. S. M., Barbour E., Al Muhayawi S. M., Al-Jaouni S., Azhar E., Qari M., Qari Y. A., Harakeh S. (2017). Saudi J. Biol. Sci..

[cit7] Official Methods of Analysis of the AOAC, ed. W. Horwitz, Maryland, USA, 17th edn, 2000

[cit8] Bogdanov S. , Harmonised methods of the International Honey Commission, Swiss Bee Research Centre, Bern, 2002

[cit9] Sakač M., Jovanov P., Marić A., Pezo L., Kevrešan Ž., Novaković A., Nedeljković N. (2019). Food Chem..

[cit10] Rufián-Henares J. A., de la Cueva S. P. (2008). Food Addit. Contam., Part A.

[cit11] Petisca C., Henriques A. R., Pérez-Palacios T., Pinho O., Ferreira I. M. P. L. V. O. (2014). J. Food Compos. Anal..

[cit12] Ariffin A. A., Ghazali H. M., Kavousi P. (2014). Food Chem..

[cit13] Tomasini D., Sampaio M. R. F., Caldas S. S., Buffon J. G., Duarte F. A., Primel E. G. (2012). Talanta.

[cit14] Ferreira I. C. F. R., Aires E., Barreira J. C. M., Estevinho L. M. (2009). Food Chem..

[cit15] Zhishen J., Mengcheng T., Jianming W. (1999). Food Chem..

[cit16] Hatano T., Kagawa H., Yasuhara T., Okuda T. (1988). Chem. Pharm. Bull..

[cit17] Karaman M., Mimica-Dukić N., Knežević P., Svirčev Z., Matavulj M. (2009). Int. J. Med. Mushrooms.

[cit18] Sun T., Ho C. T. (2001). J. Food Lipids.

[cit19] Hu X., Weng Q. (2009). Remote Sens. Environ..

[cit20] Grieu S., Faugeroux O., Traoré A., Claudet B., Bodnar J. L. (2011). Energy Build..

[cit21] Kollo T. and von Rosen D., Advanced multivariate statistics with matrices, Springer, Netherland, 2006

[cit22] Trelea I. C., Raoult-Wack A. L., Trystram G. (1997). Food Sci. Technol. Int..

[cit23] Montaño J. J., Palmer A. (2003). Neural Computing and Applications.

[cit24] Taylor B. J. , Methods and procedures for the verification and validation of artificial neural networks, Springer, New York, 2005

[cit25] Boussaid A., Chouaibi M., Rezig L., Hellal R., Donsì F., Ferrari G., Hamdi S. (2018). Arabian J. Chem..

[cit26] Gül A., Pehlivan T. (2018). Saudi J. Biol. Sci..

[cit27] Nascimento K. S. D., Gasparotto Sattler J. A., Lauer Macedo L. F., Serna González C. V., Pereira de Melo I. L., da Silva Araújo E., Granato D., Sattler A., de Almeida-Muradian L. B. (2018). Lebensmittel-Wissenschaft & Technologie.

[cit28] Bouhlali E. D. T., Bammou M., Sellam K., Ramchoun M., Benlyas M., Alem C., Filali-Zegzouti Y. (2016). J. Chem. Pharm. Res..

[cit29] Kek S. P., Chin N. L., Yusof Y. A., Tan S. W., Chua L. S. (2014). Agriculture and Agricultural Science Procedia.

[cit30] Chakir A., Romane A., Marcazzan G. L., Ferrazzi P. (2016). Arabian J. Chem..

[cit31] Kowalski S. (2013). Food Chem..

[cit32] Basheer I. A., Hajmeer M. (2000). J. Microbiol. Methods.

[cit33] Chattopadhyay P. B., Rangarajan R. (2014). Agric. Water Manag..

[cit34] Turányi T. and Tomlin A. S., Analysis of kinetic reaction mechanisms, Springer, Berlin Heidelberg, 2014

