# Supplementary material for: A comprehensive study of parameters correlated with honey health benefits
Source: RSC Adv. 2021 Mar 30;11(20):12434–41. doi: 10.1039/d0ra10887a (PMC8696875; doi:10.1039/d0ra10887a)
Supplement: RA-011-D0RA10887A-s001 [file RA-011-D0RA10887A-s001.pdf]

Electronic Supplementary Material (ESI) for RSC Advances.
This journal is © The Royal Society of Chemistry 2021

Supplementary material

A comprehensive study of parameters correlated with honey health benefits

Aleksandar Marić[a], Pavle Jovanov[a*], Marijana Sakač[a], Aleksandra Novaković[a], Miroslav Hadnađev[a], Lato Pezo[b], Anamarija Mandić[a], Nataša Milićević[a], Ana Đurović[c], Slobodan Gadžurić[d]

[a]Institute of Food Technology in Novi Sad, University of Novi Sad, Bulevar cara Lazara 1, 21000 Novi Sad, Republic of Serbia

[b]Institute of General and Physical Chemistry, University of Belgrade, Studentski trg 12-16, 11000 Belgrade, Republic of Serbia

[c]Faculty of Technology, University of Novi Sad, Bulevar cara Lazara 1, 21000 Novi Sad, Republic of Serbia

[d]Faculty of Sciences, University of Novi Sad, Trg Dositeja Obradovića 3, 21000 Novi Sad, Republic of Serbia

***Corresponding author:**

Tel. +381 21 485 3790;

Bulevar cara Lazara 1, 21000 Novi Sad, Republic of Serbia;

e-mail: pavle.jovanov@fins.uns.ac.rs

Table S1

Physicochemical parameters of different types of honey (acacia, sunflower, meadow, and forest)

Honey type		Moisture (%)	pH	Acidity (meq/kg)	Conductivity (mS/cm)	HMF (mg/kg)	Glucose (%)	Fructose (%)	Total minerals (mg/kg)
Acacia	Min	15.4	3.6	5.3	0.1	0.90	27.9	28.9	49.7
(n=25)	Max	18.7	5.8	33.5	0.3	46.0	31.8	37.4	404
	Mean	17.6^{a}	4.5^{b}	16.1^{a}	0.2^{a}	15.2^{a}	29.9^{a}	33.0^{ab}	140^{a}
	SD	0.9	0.5	6.3	0.1	11.3	0.9	2.3	101
Sunflower	Min	15.2	3.2	22.0	0.3	0.89	28.2	30.2	223
(n=25)	Max	20.3	5.0	38.5	0.5	47.8	33.9	37.2	529
	Mean	17.2^{a}	3.7^{a}	27.7^{ab}	0.4^{ab}	21.4^{a}	31.2^{a}	34.6^{b}	309^{ab}
	SD	1.4	0.6	4.2	0.1	13.1	2.3	1.8	77.8
Meadow	Min	16.2	3.6	3.5	0.0	0.25	27.8	29.1	62.6
(n=25)	Max	20.4	5.5	51.7	0.5	84.8	32.9	35.7	1073
	Mean	17.9^{a}	4.4^{b}	22.8^{ab}	0.3^{a}	27.4^{a}	30.0^{a}	32.1^{a}	527^{b}

	SD	0.9	0.4	10.4	0.1	19.8	1.19	1.72	257
Forest	Min	15.2	3.56	20.5	0.1	5.3	27.0	30.5	906
(n=25)	Max	19.1	5.23	49.3	1.4	150	33.0	37.4	2695
	Mean	17.7^{a}	4.5^{b}	38.0^{b}	0.5^{b}	30.6^{a}	29.9^{a}	33.4^{ab}	1289^{c}
	SD	1.03	0.52	5.4	0.2	27.6	2.10	2.06	367

SD – standard deviation; Min – minimum value; Max – maximum value

Means in the same column with different superscript are statistically different ($p \leq 0.05$).

Table S2

Colour parameters (*L*, *a** and *b**) of different types of honey (acacia, sunflower, meadow, and forest)

		Colour parameters		
Honey type		*L**	*a**	*b**
Acacia	Min	64.6	-4.61	6.2
(n=25)	Max	84.5	-0.91	32.0
	Mean	78.5 ^{b}	-2.65 ^{a}	18.2 ^{a}
	SD	5.05	0.80	8.2
Sunflower	Min	42.30	-2.95	26.3
(n=25)	Max	56.1	-1.76	35.2
	Mean	49.2 ^{a}	-2.55 ^{a}	30.2 ^{b}
	SD	3.29	0.24	1.6
Meadow	Min	64.2	-3.60	10.3
(n=25)	Max	81.6	-1.22	42.6
	Mean	75.3 ^{b}	-2.12 ^{a}	21.6 ^{ab}
	SD	4.59	0.60	8.8

Forest	Min	48.5	9.13	40.9
(n=25)	Max	62.3	21.06	56.4
	Mean	54.9^{a}	13.1^{b}	46.5^{c}
	SD	3.32	3.50	4.41

SD – standard deviation; Min – minimum value; Max – maximum value

Means in the same column with different superscript are statistically different ($p \leq 0.05$).

Table S3

Phenolic, flavonoid and carotenoid content, and DPPH radical scavenging activity of different types of honey (acacia, sunflower, meadow, and forest)

Honey type		Polyphenols (mg GAE/100g)	Flavonoids (mg CAE/100g)	Carotenoids (mg BCE/kg)	DPPH, IC_{50} (mg/mL)
Acacia	Min	11.1	0.99	1.24	129
(n=25)	Max	22.4	6.93	2.08	499
	Mean	16.5[a]	4.15[a]	1.67[a]	313[b]
	SD	2.51	1.40	0.23	82.7
Sunflower	Min	17.8	9.25	3.21	202
(n=25)	Max	25.8	14.0	4.21	629
	Mean	22.1[ab]	10.9[b]	3.86[c]	322[b]
	SD	1.96	1.20	0.23	72.3
Meadow	Min	9.87	0.56	1.82	127
(n=25)	Max	28.9	10.8	4.35	397
	Mean	21.3[ab]	6.14[a]	2.78[b]	260[ab]

	SD	4.36	2.21	0.67	58.3
Forest (n=25)	Min	10.3	7.99	1.84	50.2
	Max	27.8	15.2	3.63	474
	Mean	23.0[b]	11.4[b]	2.53[b]	104[a]
	SD	3.87	1.67	0.32	79.8

SD – standard deviation; Min – minimum value; Max – maximum value

Means in the same column with different superscript are statistically different ($p \leq 0.05$).

Table S4

Minimum inhibitory concentrations (MIC) of different types of honey (acacia, sunflower, meadow, and forest)against tested strains of *Bacillus subtilis*, *Enterococcus faecali*, *Staphylococcus aureu*, *Escherichia coli,Pseudomonas aeruginos* and *Proteus mirabilis*

Honey type		MIC% (v/v) against *Bacillus subtilis* ATCC 6633	MIC% (v/v) against *Enterococcus faecalis* ATCC 19433	MIC% (v/v) against *Staphylococcus aureus* ATCC 6538	MIC% (v/v) against *Staphylococcus aureus*	MIC% (v/v) against *Escherichia coli* ATCC 11229	MIC% (v/v) against *Escherichia coli* I	MIC% (v/v) against *Pseudomonas aeruginosa* ATCC 35554	MIC% (v/v) against *Proteus mirabilis* I
Acacia (n=25)	Min	9.40	18.7	2.30	9.40	18.7	18.7	18.7	18.7
	Max	18.7	18.7	4.70	18.7	37.5	37.5	37.5	37.5
	Mean	14.6^{ab}	18.7^{a}	3.26^{a}	17.2^{b}	22.5^{c}	34.5^{b}	20.2^{a}	20.2^{a}
	SD	4.71	0.0	1.20	3.48	7.68	7.03	5.21	5.21
Sunflower (n=25)	Min	9.40	18.7	2.30	18.7	18.7	37.5	37.5	37.5
	Max	18.7	18.7	2.30	18.7	18.7	37.5	37.5	37.5
	Mean	16.5^{b}	18.7^{a}	2.30^{a}	18.7^{b}	18.7^{bc}	37.5^{b}	37.5^{b}	37.5^{b}

	SD	4.05	0.00	0.00	0.00	0.00	0.00	0.00	0.00
Meadow	Min	4.70	2.30	2.30	9.40	4.70	9.40	9.40	18.7
(n=25)	Max	18.7	18.7	18.7	18.7	18.7	18.7	18.7	37.5
	Mean	11.8^{ab}	16.9^{a}	3.15^{a}	18.0^{b}	11.4^{a}	17.6^{a}	18.0^{a}	33.0^{b}
	SD	4.48	4.33	3.31	2.58	4.27	3.08	2.58	8.12
Forest	Min	4.70	9.40	2.30	9.40	9.40	9.40	18.7	9.40
(n=25)	Max	18.7	18.7	4.70	18.7	18.7	37.5	37.5	37.5
	Mean	7.50^{a}	18.3^{a}	2.78^{a}	12.0^{a}	12.0^{ab}	14.3^{a}	21.7^{a}	15.0^{a}
	SD	5.22	1.86	0.98	4.26	4.26	9.41	7.03	10.5

SD – standard deviation; Min – minimum value; Max – maximum value

Means in the same column with different superscript are statistically different ($p \leq 0.05$).

Table S5

Correlation matrix of the observed variables

	V3	V4	V5	V6	V7	V8	V9	V10	V11	V12	V13	V14	V15	V16	V17	V18	V19	V20	V21	V22
V1	-0.025	-0.183	-0.199**	-0.462^{+}	-0.289*	0.187	0.373^{+}	0.181	-0.029	-0.152	-0.014	0.087	-0.179	-0.011	-0.279*	-0.452^{+}	-0.386^{+}	0.451^{+}	0.310*	0.477^{+}
V2	0.669^{+}	0.707^{+}	0.786^{+}	0.177	-0.475^{+}	0.657^{+}	-0.606^{+}	0.636^{+}	0.697^{+}	-0.489^{+}	-0.094	-0.175	-0.498^{+}	-0.471^{+}	-0.412^{+}	0.058	-0.229**	-0.496^{+}	0.443^{+}	0.085
V3		0.711^{+}	0.752^{+}	0.265*	-0.460^{+}	0.629^{+}	-0.518^{+}	0.413^{+}	0.592^{+}	-0.416^{+}	-0.149	-0.090	-0.422^{+}	-0.446^{+}	-0.406^{+}	0.090	-0.154	-0.536^{+}	0.380^{+}	0.015
V4			0.714^{+}	0.352^{+}	-0.319*	0.518^{+}	-0.453^{+}	0.292*	0.495^{+}	-0.437^{+}	-0.211**	-0.200**	-0.341**	-0.528^{+}	-0.382^{+}	0.068	-0.043	-0.699^{+}	0.359^{+}	-0.086
V5				0.508^{+}	-0.386^{+}	0.551^{+}	-0.831^{+}	0.496^{+}	0.740^{+}	-0.316*	-0.002	-0.224**	-0.364^{+}	-0.322*	-0.194	0.393^{+}	-0.032	-0.778^{+}	0.193	-0.256**
V6					0.100	0.015	-0.634^{+}	-0.112	0.235**	0.193	0.095	-0.132	0.188	-0.122	0.146	0.618^{+}	0.549^{+}	-0.803^{+}	-0.346^{+}	-0.689^{+}
V7						-0.691^{+}	0.221**	-0.715^{+}	-0.571^{+}	0.457^{+}	0.095	0.007	0.616^{+}	0.347^{+}	0.602^{+}	0.282*	0.425^{+}	-0.106	-0.590^{+}	-0.514^{+}
V8							-0.369^{+}	0.824^{+}	0.743^{+}	-0.604^{+}	-0.073	-0.088	-0.632^{+}	-0.541^{+}	-0.690^{+}	-0.219**	-0.442^{+}	-0.188	0.666^{+}	0.419^{+}
V9								-0.430^{+}	-0.675^{+}	0.053	-0.143	0.199**	0.213**	0.142	-0.026	-0.635^{+}	-0.107	0.742^{+}	0.064	0.437^{+}
V10									0.744^{+}	-0.529^{+}	0.063	-0.037	-0.650^{+}	-0.386^{+}	-0.588^{+}	-0.176	-0.555^{+}	-0.028	0.616^{+}	0.464^{+}
V11										-0.409^{+}	0.018	-0.087	-0.471^{+}	-0.338*	-0.374^{+}	0.111	-0.319*	-0.397^{+}	0.391^{+}	0.096
V12											0.078	0.305*	0.687^{+}	0.629^{+}	0.642^{+}	0.517^{+}	0.587^{+}	0.032	-0.869^{+}	-0.658^{+}
V13												-0.538^{+}	0.056	0.157	0.228**	0.180	-0.057	0.005	-0.171	-0.130
V14													0.145	0.250**	0.006	-0.032	0.097	0.216**	-0.169	-0.018
V15														0.456^{+}	0.698^{+}	0.380^{+}	0.641^{+}	-0.011	-0.843^{+}	-0.660^{+}
V16															0.699^{+}	0.398^{+}	0.221**	0.244**	-0.682^{+}	-0.402^{+}
V17																0.535^{+}	0.392^{+}	-0.062	-0.835^{+}	-0.681^{+}
V18																	0.626^{+}	-0.578^{+}	-0.716^{+}	-0.859^{+}
V19																		-0.352^{+}	-0.749^{+}	-0.765^{+}
V20																			0.152	0.641^{+}
V21																				0.856^{+}

$^{+}$Correlation is statistically significant at $p < 0.001$ level; *correlation is statistically significant at $p < 0.01$ level; **correlation is statistically significant at $p<0.05$ level.

V1 – pH; **V2** – Free acidity; **V3** – Conductivity; **V4** – Phenols; **V5** – Flavonoids; **V6** – Carotenoids; **V7** – DPPH; **V8** – Total minerals; **V9** – *L**; **V10** – *a**; **V11** – *b**; **V12** –MIC% (v/v) against *Bacillus subtilis* ATCC 6633; **V13** – MIC% against *Enterococcus faecalis* ATCC 19433; **V14** – MIC% against *Staphylococcus aureus* ATCC 6538; **V15** – MIC% against *Staphylococcus aureus*; **V16** – MIC% against *Escherichia coli* ATCC 11229; **V17** – MIC% against *Escherichia coli* I; **V18** – MIC% against *Pseudomonas aeruginosa* ATCC 35554; **V19** – MIC% against *Proteus mirabilis* I; **V20** – RACI; **V21** – RAI; **V22** – POWER.
